# Quality of Male and Female Medical Content on English-Language Wikipedia: Quantitative Content Analysis

**DOI:** 10.2196/47562

**Published:** 2024-09-12

**Authors:** Nuša Farič, Henry WW Potts, James M Heilman

**Affiliations:** 1 School of Informatics University of Edinburgh Edinburgh United Kingdom; 2 Advanced Care Research Center Usher Institute University of Edinburgh Edinburgh United Kingdom; 3 University College London Institute of Health Informatics London United Kingdom; 4 Department of Emergency Medicine Faculty of Medicine University of British Columbia Vancouver, BC Canada

**Keywords:** Wikipedia, wikis, writing, internet, health information, sex, sex bias, consumer health information, health communication, public education, social media

## Abstract

**Background:**

Wikipedia is the largest free online encyclopedia and the seventh most visited website worldwide, containing >45,000 freely accessible English-language medical articles accessed nearly 1.6 billion times annually. Concerns have been expressed about the balance of content related to biological sex on Wikipedia.

**Objective:**

This study aims to categorize the top 1000 most-read (most popular) English-language Wikipedia health articles for June 2019 according to the relevance of the article topic to each sex and quality.

**Methods:**

In the first step, Wikipedia articles were identified using WikiProject Medicine Popular Pages*.* These were analyzed on 13 factors, including total views, article quality, and total number of references. In the second step, 2 general medical textbooks were used as comparators to assess whether Wikipedia’s spread of articles was typical compared to the general medical coverage. According to the article’s content, we proposed criteria with 5 categories: 1=“exclusively female,” 2=“predominantly female but can also affect male individuals,” 3=“not sex specific or neutral,” 4=predominantly male but can affect female individuals,” and 5=“exclusively male.”

**Results:**

Of the 1000 Wikipedia health articles, 933 (93.3%) were not sex specific and 67 (6.7%) were sex specific. There was no statistically significant difference in the number of reads per month between the sex-specific and non–sex-specific articles (*P*=.29). Coverage of female topics was higher (50/1000, 5%) than male topics (17/1000, 1.7%; this difference was also observed for the 2 medical textbooks, in which 90.2% (2330/2584) of content was not sex specific, female topics accounted for 8.1% (209/2584), and male topics for accounted for 1.7% (45/2584; statistically significant difference; Fisher exact test *P*=.03). Female-category articles were ranked higher on the Wikipedia medical topic importance list (top, high, or mid importance) than male-category articles (borderline statistical significance; Fisher exact test *P*=.05). Female articles had a higher number of total and unique references; a slightly higher number of page watchers, pictures, and available languages; and lower number of edits than male articles (all were statistically nonsignificant).

**Conclusions:**

Across several metrics, a sample of popular Wikipedia health-related articles for both sexes had comparable quality. Wikipedia had a lower number of female articles and a higher number of neutral articles relative to the 2 medical textbooks. These differences were small, but statistically significant. Higher exclusively female coverage, compared to exclusively male coverage, in Wikipedia articles was similar to the 2 medical textbooks and can be explained by inclusion of sections on obstetrics and gynecology. This is unlike the imbalance seen among biographies of living people, in which approximately 77.6% pertain to male individuals. Although this study included a small sample of articles, the spread of Wikipedia articles may reflect the readership and the population’s content consumption at a given time. Further study of a larger sample of Wikipedia articles would be valuable.

## Introduction

### Background

Wikipedia is the largest, free, multilingual online encyclopedia. It is the seventh most-visited website worldwide and is available in 329 languages, with 6,829,103 articles in the English language as of May 30, 2024 [1-3]. It is also a source for a new generation of artificial intelligence technologies, such as ChatGPT [4]. Wikipedia articles can be edited and created by anyone from around the world.

Wikipedia includes >45,000 freely accessible English articles related to medical topics, which are accessed nearly 1.8 billion times per year [5]. The editors of health articles range from physicians, researchers, policy makers, medical students, and anyone else interested in human health (eg, patients) [6,7]. Medical articles include topics about human health, health organizations, and notable people in medicine and health care. On Wikipedia, these articles are ranked according to their importance to the field of medicine, and they are also ranked in order of popularity in terms of number of views (traffic to the page). These articles are also assessed for quality within 6 categories ranging from “stub” (an article with limited information, not considered enough as an encyclopedic coverage) to “start,” “C-class,” “B-class,” “good article” (GA), and “featured article” (FA; with an in-depth examination and peer review) [8]. Quality can also be assessed by a language-agnostic quality prediction tool that can predict the quality of the Wikipedia article using a single model based on length, references, images, categories, links, and sections [9].

Before COVID-19, estimates of English medical monthly page views on Wikipedia were around 200 million, with the early part of the pandemic in March 2020 seeing a rise to 400 million hits [10]. This number decreased to around 150 million in January 2022 [10]. Estimates in December 2021 found that Wikipedia was the fourth most-visited website for health content (approximately 260 million page views across all languages), surpassing WebMD (approximately 240 million page views), the Mayo Clinic (approximately 230 million page views), the World Health Organization (approximately 80 million page views), and UpToDate (approximately 50 million page views), but below Healthline (which had the highest level of hits; approximately 1100 million page views), the US National Institutes of Health (approximately 600 million page views), and the United Kingdom National Health Service (approximately 460 million page views) [10]. Nonetheless, Wikipedia remains among the most widespread resources for accessing health or medical information.

### Online Health-Seeking Behavior

The most preferred way of obtaining health information is seeking it on the web because of fewer barriers to accessing content, affordability, coverage of information, interactivity, privacy, and confidentiality [11,12]. Information seeking is often initiated as a response to uncertainty regarding one’s health, such as considering treatment options or as preparation for a consultation with a health care provider [13,14]. Among other factors, sex has been shown to play a considerable role in information-seeking behavior in some studies, suggesting that female individuals are more active seekers of general health-related information than male individuals, even across disparities in health literacy [15]. However, other studies have not confirmed any difference in health information seeking between the sexes (eg, [13]) and instead argue that this is likely because of sociodemographic factors and individual differences. Health information is often also subject to scrutiny from both health care professionals and laypeople [16]. Part of health behavior, either by default or as a rule, involves seeking and evaluating the quality and reliability of information presented to us [16].

### Representativeness of Male or Female Wikipedia Content

Wikipedia editors have been reported to be predominantly male for medical and health articles [7], and studies have found similar trends for other topics on Wikipedia in different languages, with about 85% to 90% of editors identifying as male, 10% to 15% as female, and 1% to 2% as transgender, even after a slow increase in female contributors from year to year [17-21], a difference attributed to the enduring gender imbalance in computer-related fields [22]. This difference in editing numbers among the self-identified sexes is believed to be due to psychosocial factors [23,24]. Some editorial behavior is influenced by Wikipedia culture across the whole encyclopedia and the tensions between different editors (eg, core contributors vs peripheral contributors) [25]. If this study finds obvious unequal coverage for either sex, it is a possibility that it may partly be explained by the creators of the content [26].

Disparity in gender representation and participation in technology-related fields, particularly in open-source communities, has been studied since the 1980s [22]. There is sufficient evidence that gender imbalance carries into content generation via social contexts because of various factors, such as women taking up fewer technology-related jobs and sociocultural factors surrounding women (eg, gender stereotypes, gender expectations, women being averse to conflict, lack of time, other life responsibilities such as raising children, lack of user-friendliness, women steering away from competitive environments, women fearing that their content would be deleted or reverted [“impostor syndrome”], and personal choice) [24,27]. The evidence for sexism on Wikipedia is mixed. One article states, “WP [Wikipedia] is male dominated. That doesn’t mean it is sexist” [28]. Most Wikipedia editor profiles are anonymous (ie, use a username rather than own identity), and it would be difficult to enact direct sexism; however, some reports showed Wikipedia being hostile to women (eg, female editors being harassed by male colleagues) [29]. Studies have revealed that Wikipedia is biased in how men and women are characterized in their biographies [30], including how women’s biographies suitable for inclusion on Wikipedia are twice as frequently categorized into nonnotable and are more frequently nominated for deletion (25%) than men’s biographies (17%) [31]. This is true even after international coverage of the “Donna Strickland effect”—a female Nobel Prize winner in physics in 2018 whose Wikipedia profile was deleted [31]. This points to multilayered gender discrimination and may carry over into content production [32]. Considering this, no studies have been performed exclusively on health and medical content related to sex and gender.

Despite fewer women than men editing Wikipedia, recent research found a minimal amount of evidence of biological sex bias in terms of content [33,34], and 1 study found evidence of bias against men, with articles on male chief executive officer (CEO) profiles generally having a longer profile life span but less reliable sources, being less edited, and receiving less attention than female profiles [35]. Notably, for coverage of biographies of living persons, out of 1.7 million biographies on Wikipedia, as of May 5, 2019, a total of 77.6% were of men and 22.7% were of women, pointing to possible inequalities in digital coverage of biographies of people [36]. Other researchers reported the proportion of female biographies to be at 16% [33].

Several studies have also looked into the gender distribution gap in the representation of actual social groups [37]. For example, a study of actively publishing economists on Wikipedia found that women are half as likely to have a biographical entry on the English-language Wikipedia than men [38]. Halfaker [39] found that for English-language Wikipedia, the average quality of articles on women scientists was lower than that of male scientists before 2014 but exceeded the average quality after that. A study on women mathematicians found biases toward men [40], whereas a study including Fortune’s top 1000 CEO profiles found a bias toward women [41]. It is important to note that these disparities likely reflect the traditional sex roles that existed in society before the turn of the century.

There are recent efforts by Wikipedia to remove this gap (#WikiGap and #ProjectRewrite, particularly by Wikimedia Sweden) and by WikiProject Women in Red to address sex inequality on Wikipedia [42-44]. Despite the still existing gaps in the coverage of biographies of living persons, it is unclear if the same disparity exists within other topic areas of Wikipedia, for example, the medical and health pages or regarding content coverage of female or male health topics [45,46]. Because anyone can benefit from access to medical and health information and because health and medical information is available in hundreds of languages, we decided to investigate this area for clarification. We are not investigating the number of male and female editors or readers but are specifically interested in the coverage of male and female health content on Wikipedia. There is a lack of research about the coverage of sex-based health and medical topics in the English-language Wikipedia. We also wanted to compare the Wikipedia articles to the coverage of the topics in general medical textbooks, which, in this study, will provide our baseline.

Access to free, reliable, and up-to-date health information is essential because of the size of global traffic to medical and health pages, including Wikipedia. If we learn that there is an obvious disparity in the coverage of medical and health topics, we may be able to investigate the possible contributing factors. We would also like to identify Wikipedia article types that may require improvement in terms of quality.

### Objectives

Because of Wikipedia’s significance as a leading global resource for health information, this study aims to explore potential disparities in the coverage of health articles related to biological sex. The objectives of the study are as follows: (1) to analyze Wikipedia’s top 1000 most-read health articles and determine the coverage of sex-specific and non–sex-specific topics, and (2) to assess the quality of male versus female health content on English-language Wikipedia, using both Wikipedia’s classification system and other markers of completeness.

## Methods

### Ethical Considerations

The data that we extracted from Wikipedia is freely available. It did not include any personally identifiable data or any other forms of data that may cause a breach of any ethical or regulatory rules and regulations. No ethics approval was needed.

### Design

This study used quantitative research methods. We have concentrated on the top 1000 Wikipedia health articles as the most popular Wikipedia pages by traffic.

### Search Strategy

First, we defined what we meant by “male” and “female” health topics. We focused on the medical, biological, or physical characteristics of the human body and not gender (social perception and individual choice of identity). We defined male articles in which the Wikipedia topic in question included at least 80% of people with male biological sex, female articles in which the Wikipedia topic in question included at least 80% of people with female biological sex, and not sex specific, in which the Wikipedia topic in question could relate in equal proportions to all biological sexes. Therefore, we developed criteria for sex-specific and non–sex-specific health articles based on the degree to which they may correspond to sex specificity: 1=exclusively female, 2=predominantly female but can affect male individuals, 3=not sex specific or neutral, 4=predominantly male but can also affect female individuals, 5=exclusively male. Second, a list of the 1000 most-read medical articles on Wikipedia for June 2019 was identified using the “WikiProject Medicine Popular pages” list, which is a list of the top 1000 pages ordered by the number of views as of July 16, 2019 [5] (Wikipedia provides an overview of top articles monthly; hence, we collected data for June 2019 in July 2019).

### Eligibility Criteria

Our research was not guided by any framework, although similar categories have been analyzed in previous Wikipedia literature [14]. Topics related to female anatomy and childbirth and conditions that affect female individuals were deemed “exclusively female.” Articles related to male anatomy were deemed to be “exclusively male.” Non–sex-specific articles included topics such as famous people in medicine (both physicians and patients alike, eg, Louis Pasteur, Florence Nightingale, and Phineas Gage), topics ranging from epidemiology or political events (eg, rape statistics and domestic violence), lifestyles related to health (eg, Mediterranean diet, noise pollution, and surrogacy), medical procedures (eg, Rorschach test, hygiene, and x-ray), medical drugs and chemical compounds (eg, buprenorphine, vitamin E, and cannabis), poisonous plants (eg, Datura), medical literature (eg, PubMed and the *Diagnostic and Statistical Manual of Mental Disorders*), medical conditions (eg, hernia, gastritis, and polio), medical terminology (eg, VO_2_ max, biosafety level, and reflex syncope), medical events (eg, 2019 India doctor’s strike and National Doctor’s Day), and medical professions (eg, pathology and nursing).

### Screening Process

Our methodology included several steps. After extracting a list of the 1000 most-read medical articles on Wikipedia for June 2019, the authors extracted only sex-specific articles (n=67, 6.7%) and placed them in a spreadsheet (Multimedia Appendix 1). The remaining articles concerned health or medical issues previously mentioned in various neutral categories and were therefore classified as “not sex specific.” The list of medical articles was reviewed manually by 2 authors (JMH and NF) to classify the article as primarily or exclusively related to health care in either sex. Disagreements were resolved via discussion and by searching peer-reviewed medical publications for existing data to resolve unclear cases.

### Assessment of the Quality of Articles

We needed to create a comparator to conclude whether the coverage of medical topics on Wikipedia was typical or deviated from a gold standard for the general medical field. Therefore, in step 2, we followed the same method of classification as in step 1, but we replaced the source with medical textbooks. We chose 2 medical textbooks and evaluated topics in the same way as in step 1. Medical textbooks that are standardly used in medical or medicine studies in the United Kingdom and Canada, where the authors are based, are the *Oxford Handbook of Clinical Medicine* [47] and *Toronto Notes: Comprehensive Medical Reference and Review for Medical Council of Canada Qualifying Examination and US Medical License Examination* [48]. The counts for these were kept in the Excel spreadsheet for each separate resource (Tables 1 and 2). Unlike in step 1, we did not assess the quality of medical content in the medical textbooks.

**Table 1:** Breakdown of the results for sex-specific health articles on Wikipedia in terms of article assessment.

Number of Wikipedia articles based on article assessment	FA^a^, n (%)	GA^b^, n (%)	B^c^, n (%)	C^d^, n (%)	Start^e^, n (%)	Total, n (%)
Male	0 (0)	1 (6)	7 (41)	6 (35)	3 (18)	17 (100)
Female	2 (4)	2 (4)	32 (64)	10 (20)	4 (8)	50 (100)

^a^FA: featured article status.

^b^GA: good article status.

^c^B: B-class article status.

^d^C: C-class article status.

^e^Start: “start” article status.

**Table 2:** Breakdown of the results for sex-specific health articles on Wikipedia in terms of article status.

Importance rating of male vs female health-related Wikipedia articles	Top, n (%)	High, n (%)	Mid, n (%)	Low, n (%)	Total, n (%)
Male	1 (6)	3 (18)	10 (59)	3 (18)	17 (100)
Female	7 (14)	23 (46)	17 (34)	3 (6)	50 (100)

### Categories Generated

We developed 5 categories: 1=“exclusively female,” 2=“predominantly female but can also affect male individuals,” 3=“not sex specific or neutral,” 4=predominantly male but can affect female individuals,” and 5=“exclusively male.” Category 2, for example, covers urinary tract infection, breast cancer, human papillomavirus, anorexia, and bulimia nervosa, which all primarily affect the female population, but they can also affect male individuals. Similarly, there are conditions or topics concerning health that primarily affect male individuals but can be found in female individuals too, such as testosterone or inguinal hernia [49], which make up category 4. Our cutoff point for “predominantly” categories was 80% of cases (eg, inguinal hernia appears in an 8:1 male:female ratio) [49].

### Statistical Analyses

To assess statistically significant differences for probability samples, we performed standard nonparametric tests, including the Mann-Whitney test and Fisher exact tests. Mann-Whitney tests were chosen as our outcome variables typically do not follow a normal distribution, and Mann-Whitney tests make few distributional assumptions. Fisher exact tests are preferred for contingency tables as they provide an exact test, especially because smaller sample sizes can lead to low expected cell counts [50].

## Results

### Overview

Concerning the first aim, 67 sex-specific Wikipedia articles were extracted and are displayed in the Multimedia Appendix 1. For each article, the table includes information on the 13 items: rank by number of page views, title of the page, number of page views that month, WikiProject Medicine’s quality assessment, WikiProject Medicine’s assessment of importance to medicine, classification, bytes of text, unique references, bytes of text per reference, number of pictures, number of sections, total edits, and number of page watchers (Wikipedia editors who have the page on their watchlist).

### Breakdown of Health Articles

Of the 67 sex-specific medical articles on Wikipedia, 17 (25%) concerned male individuals or predominantly male individuals (exclusively male: n=9, 53%; predominantly male: n=8, 47%), while 50 (75%) concerned female individuals or predominantly female individuals (exclusively female: n=37, 74%; predominantly female: n=13, 26%). The remaining 93.3% (933/1000) of health and medical articles were either unrelated to diseases and medications or were classified as neutral or not sex specific. For quality assessments, male and female articles were grouped to include the predominant and exclusive categories (ie, 1 and 2 vs 4 and 5). When examining Wikipedia articles by type of article, we recognized that 8% (80/1000) of the articles were about political events, people, or medical terminology (eg, the black death, the scientific method, and Theranos) that are generally not covered in medical textbooks.

### Assessment of Wikipedia Articles or Their Article Status

#### Overview

We looked at 1000 out of 50,000 health-related Wikipedia articles which represent 2% of all health content on the English-language Wikipedia, however, these articles made up 43% of total page views (65/155 million) in June 2019. Of the 67 sex-specific articles, the mean average daily views for all sex-specific health articles were 1968 (SD 975.1) views per day, and the mean of total views was 58,877.9 (SD 29,251). Of the 67 sex-specific articles, 2 (3%) were classified as FA status, 3 (5%) as GA, 39 (58%) as B-class, 13 (19%) as C-class, and 7 (10%) as “start.” In terms of article rating, slightly more female articles than male articles had FA status (0/67, 0% male articles; 2/67, 4% female articles) and a B-class status (7/67, 41% male articles; 32/67, 64% female articles); however, the number of male articles within GA status (1/67, 6% male articles; 2/67, 4% female articles), C-class (6/67, 35% male articles; 10/67, 20% female articles), and “start” (3/67, 18% male articles; 4/67, 8% female articles) was slightly higher than that for female articles (Table 1). This was not a statistically significant difference (Fisher exact *P*=.29). FA was only found for exclusively female articles, which included “female genital mutilation” and “menstrual cycle.” The GA class in exclusively male or predominantly male Wikipedia articles included “circumcision,” whereas for the exclusively female and predominantly female articles, GA articles included “birth control” and “urinary tract infection.” The start category included “priapism,” “minoxidil,” and “penis enlargement” for exclusively male or predominantly male articles and “gynecology,” “Braxton Hicks contractions,” “menstrual cup,” and “triple X syndrome” for exclusively female or predominantly female articles.

#### Wikipedia Importance Rating

Rank place” is the order of the article based on the number of visits and reads per month, with higher viewed articles being higher on the list. The sex-specific articles ranged from rank place 30 to rank place 929 on the list of the 1000 most visited medical articles on Wikipedia. They do not differ significantly by rank (Mann-Whitney test, *Z*=0.25; *P*=.81).

Proportionally, a higher number of female health articles were classified as top priority (1/67, 6% male articles; 7/67, 14% female articles) and high priority (3/67,18% male articles; 23/67, 46% female articles) but not mid (10/67, 59% male articles; 17/67, 34% female articles) and low priority (3/67, 18% male articles; 3/67, 6% female articles; Table 2). This result was borderline for statistical significance (Fisher exact *P*=.05).

#### Wikipedia Total References

The number of total references for the whole sample was 10,968 references. For female category articles, the total number of references was 8788, whereas for male category articles, this was 1918. This amounts to a mean of 175.8 references per article for the exclusively female or predominantly female category and 128.2 references per article for the exclusively male or predominantly male category. This difference is not statistically significant (*Z*=1.7; *P*=.08).

#### Wikipedia Unique References

The number of unique references for the whole sample was 6715. For female category articles, this was 5308, and for male category articles, unique references totaled 1407. This amounts to a mean of 106.2 unique references per article for the exclusively female or predominantly female category and 82.7 unique references per article for exclusively male or predominantly male categories. This is not a statistically significant difference (*Z*=1.4; *P*=.18).

#### Wikipedia Number of Edits

The total edit number for all 67 sex-specific Wikipedia health articles was 188,541, of which 130,522 (69.2%) were for female category articles and 58,019 (30.7%) were for male category articles. This amounts to a mean of 2610 for female-category articles and 3413 for male-category articles. This is not a statistically significant difference (*Z*=1.4; *P*=.18).

#### Wikipedia Watchers of Health Articles

The total number of page watchers for all Wikipedia health articles was 16,426. For female category articles, the total number of page watchers was 11,692, whereas for male category articles, the total number of page watchers was 4735. This is a mean of 234 watchers per female category pages and 279 watchers for male category pages. This is not a statistically significant difference (*Z*=1.1; *P*=.27).

#### Wikipedia Article Pictures

The total number of pictures in all Wikipedia health articles was 799. For female category articles, the total number of pictures was 613, whereas for male category articles, the total number of pictures was 186. This average is 12.3 pictures per article for female category articles and 10.9 pictures per article for male category articles. This is not a statistically significant difference (*Z*=.34; *P*=.73).

#### Language Count

We have assessed the differences regarding the number of languages in which sex-specific Wikipedia articles were available. The Mann-Whitney test showed no statistical significance between the sex-specific article categories (*P*=.36). Language count was slightly higher for female category articles (mean 55.9 languages) than male category articles (mean 48.4 languages).

#### Breakdown of the 2 Medical Textbooks

The comparator table (Table 3) includes the breakdown of typical or general coverage of the 2 medical textbooks as comparators to Wikipedia. The coverage was similar. For the *Oxford Handbook of Clinical Medicine* [47], the coverage was 1.2% (7/560) exclusively female, 4.2% (24/560) predominantly female, 93.4% (522/560) neutral or not sex specific, 0.7% (4/560) predominantly male, and 0.5% (3/560) exclusively male. For *Toronto Notes Medical Council of Canada Qualifying Examination and United States Medical License Examination II* [48], the coverage was 6.9% (139/2024) exclusively female, 1.9% (39/2024) predominantly female, 89.3% (1808/2024) neural or not sex specific, 0.8% (16/2024) predominantly male, and 1.1%% (22/2024) exclusively male. There was a statistically significant difference when comparing Wikipedia to the combined counts across both textbooks (Fisher exact *P*=.03) with Wikipedia having a lower number of female health articles but a higher number of neutral articles than the 2 medical textbooks. However, the absolute differences were not large. Table 4 shows a comparison of final results by each of the 3 sources (Wikipedia, book 1, and book 2), with an additional row for combined book 1 and book 2, for those who may be interested in comparing Wikipedia to the 2 textbooks together.

**Table 3:** Breakdown of sex-specific topics for the 2 medical textbooks as comparators to Wikipedia health articles’ coverage.

Books and topics listed within the book			Number of topics^a^								
			1		2		3		4		5
***Oxford Handbook of Clinical Medicine*** **[47] (n=560)**											
	Thinking about medicine	1		0		16		0		0	
	History and examination	0		0		23		0		0	
	Cardiovascular medicine	0		0		30		0		0	
	Chest medicine	0		0		22		0		0	
	Endocrinology	2		0		23		1		1	
	Gastroenterology	0		2		34		1		0	
	Renal medicine	1		1		12		0		0	
	Hematology	0		3		30		0		0	
	Infectious diseases	0		0		33		0		0	
	Neurology	0		2		38		1		0	
	Oncology and palliative care	1		11		6		1		0	
	Rheumatology	0		1		16		0		0	
	Surgery	1		3		52		0		2	
	Clinical chemistry	1		1		24		0		0	
	Eponymous syndromes	0		0		90		0		0	
	Radiology	0		0		16		0		0	
	Reference intervals, etc	0		0		5		0		0	
	Practical procedures	0		0		10		0		0	
	Emergencies	0		0		42		0		0	
	Total, n (%)	7 (1.2)		24 (4.2)		522 (93.4)		4 (0.7)		3 (0.5)	
***Toronto Notes: Comprehensive Medical Reference and Review for Medical Council of Canada Qualifying Examination and US Medical License Examination II*** **[48] (n=2024)**											
	Ethical, legal, and organizational medicine	0		1		20		0		0	
	Anesthesia and perioperative medicine	0		0		58		0		0	
	Cardiology and cardiac surgery	0		1		62		1		0	
	Clinical pharmacology	0		0		24		0		0	
	Dermatology	0		0		77		0		0	
	Emergency medicine	0		1		71		0		0	
	Endocrinology	1		3		70		2		0	
	Family medicine	0		5		59		1		6	
	Gastroenterology	1		4		86		1		0	
	General surgery and thoracic surgery	0		2		105		2		0	
	Geriatric medicine	0		4		20		3		0	
	Gynecology	53		0		1		0		0	
	Hematology	0		1		80		0		0	
	Infectious diseases	0		0		90		0		0	
	Medical genetics	0		1		9		0		0	
	Medical imaging	0		0		52		0		0	
	Nephrology	0		0		55		0		0	
	Neurology	0		1		99		0		0	
	Neurosurgery	0		0		65		1		0	
	Obstetrics	82		1		0		0		0	
	Ophthalmology	0		1		107		0		0	
	Orthopedic surgery	0		0		96		0		0	
	Otolaryngology	0		4		87		0		0	
	Pediatrics	0		2		108		1		0	
	Plastic surgery	0		1		58		0		0	
	Psychiatry	0		0		87		0		0	
	Public health and preventive medicine	1		2		44		0		0	
	Respirology	1		1		47		0		0	
	Rheumatology	0		2		37		0		0	
	Urology	0		0		23		4		16	
	Vascular surgery	0		1		11		0		0	
	Total, n (%)	139 (6.9)		39 (1.9)		1808 (89.3)		16 (0.8)		22 (1.1)	

^a^Classification category: 1=“exclusively female,” 2=“predominantly female but can also affect male individuals,” 3=“not sex specific or neutral,” 4=predominantly male but can affect female individuals,” and 5=“exclusively male.”

**Table 4:** Comparator table for topics for all 3 sources (Wikipedia and the 2 medical textbooks).

Source	Total of all articles, n (%)	Sex-specific total, n (%)	Number of topics per classification^a^, n (%)				
			1	2	3	4	5
Wikipedia	1000 (100)	67 (6.7)	37 (3.7)	13 (1.3)	933 (93.3)	8 (0.8)	9 (0.9)
Book 1: *Oxford Handbook* [47]	560 (100)	28 (5)	7 (1.2)	24 (4.2)	522 (93.4)	4 (0.7)	3 (0.5)
Book 2: *Toronto Notes* [48]	2024 (100)	216 (10.7)	139 (6.9)	39 (1.9)	1808 (89.3)	16 (0.8)	22 (1.1)
Book 1 and book 2 combined	2584 (100)	244 (9.4)	146 (5.6)	63 (2.4)	2330 (90.2)	20 (0.8)	25 (1)
Total for all 3	3584 (100)	311 (8.6)	183 (5.1)	76 (2.1)	3263 (91.1)	28 (0.8)	34 (0.9)

^a^Classification category: 1=“exclusively female,” 2=“predominantly female but can also affect male individuals,” 3=“not sex specific or neutral,” 4=predominantly male but can affect female individuals,” and 5=“exclusively male.”

## Discussion

### Principal Findings

In the top 1000 health Wikipedia articles in our selected timeframe (up to June 2019), there are almost 3 times as many exclusively female or predominantly female articles as exclusively male or predominantly male health articles. However, many of the articles contain sex ratios such as 4:1 and 5:1 (eg, “human papillomavirus” and “anabolic steroid”) as they were classified in the predominantly categories. This study uncovered that exclusively female and predominantly female articles rank higher on the priority list on Wikipedia, which could be explained by the inclusion of topics related to childbirth and gynecology. There are many more topics for the female reproductive system than for the male reproductive system. This is just the nature of biology, with the menstrual cycle, pregnancy, maternity, gynecology, and perinatal concerns alone having many more subcategories than those concerning the male reproductive system. Wikipedia health content was similar to the 2 medical textbooks, but there was a statistically significant difference, with the 2 medical textbooks having a higher number of female articles but fewer neutral articles. It is important to note that the 2 medical textbooks do not include the same degree of topics about psychology, people in medicine, or political events related to health. When examining Wikipedia articles by type of article, we recognized that 8% (80/1000) were about political events or people.

Our analysis has shown that exclusively female or predominantly female articles in this sample contained more references and more unique references than exclusively male or predominantly male health articles but with no statistically significant difference. Both categories of articles had a comparable number of pictures per article. Slightly more edits, on average, were made to exclusively male and predominantly male articles compared to the female topic categories. Both sets of articles include a single controversial article that attracted a much higher number of edits: “circumcision” and “abortion.” These 2 articles also have much higher numbers of page watchers. However, with or without these 2 articles, the number of edits and page watchers was not statistically significantly different. The controversial nature of a topic affects the number of edits to an article, making it an unreliable marker of quality. This cannot be used as a measure against a bias toward either sex but as an indication of the controversy within the subject matter.

One of the other important facts is that we did not assess readership, as it would be difficult to assess the sex of the readers, and therefore, we cannot draw any conclusions regarding the sexes of readers of specific health articles. It is possible that readership is equal between the sexes or that it varies depending on different countries, cultures, or times of the year (eg, medical school examinations).

### Comparison With Prior Work

Differences between the coverage and quality of content on Wikipedia are not new, and certainly, there are particular gaps that begin with the fact that only about 16% of Wikipedia contributors are women [17,18]. Wikipedia includes a list of all up-to-date research on the subject of sex, gender, gender gap, and women on Wikimedia projects by individuals or organizations associated with the Wikimedia Foundation (WWC2023) and external researchers [51]. The list on Gender Bias On Wikipedia categorizes types of gender bias into “content,” “participation,” and “consumption,” and includes research spanning from 2011 to 2023. This list currently includes 42 research projects for “content,” 17 items for “participation,” and 3 items for “consumption.” This study will likely fit under the “content” gender bias type. Of these 42 studies, only 1 (2%; on measuring what matters in gender diversity) mentions health content on Wikipedia [52]. This study cautions us on using reader attention as a proxy for the importance of the content, given the consistent gender gap in readership and self-focus bias (eg, men reading more biographies of men compared to women), further recognizing that health articles are not biographies and have clear gender dimensions [52].

A study in 2020 on metrics for quantifying the gender content gap on Wikipedia has shown that in some instances, such as in health care, gender division is important because it allows Wikipedia to organize efforts to improve the gender gap [51]. We found that on Wikipedia female and male sex categories were comparable in quality, but not in spread. Female category articles were more numerous and also had a higher (albeit nonstatistically significant) number of references and unique references and a higher number of articles in FA status. Wikipedia also contained less content on female health than the 2 medical textbooks. Our results are somewhat in line with a study conducted in 2015 that compared the mean length of the articles on men and women for English-language Wikipedia, in which articles on women were longer (statistically significant) than those on men [30]. A study in 2016 also found that articles on female CEOs had more references, which were more diverse [41]. This contrasts with the findings of Gray [36] and Konieczny and Klein [53], who, across 25 different language versions of Wikipedia, found that articles about women were consistently 10% shorter on average than those about men.

Although our articles ranked similarly on quality for male-specific and female-specific topics, this does not mean there is no room for improvement. For example, all articles, particularly those in health care and medicine, have the potential to reach FA status by improving on several quality dimensions. However, we should remain mindful that the completeness of content may reflect global social or demographic aspects. Articles on certain topics could be needed more because of sociodemographic reasons and not because of favorability or exclusion of one sex over the other.

Our study should also not downplay the fact that there could still be other types of inequalities within the content that we could not assess (eg, language structure). For example, a 2019 study found that women in physics, economics, and philosophy are considerably less likely than men to be recognized on Wikipedia across all levels of achievement [54,55]. Differences have been observed in metadata, language, and network structure when comparing male and female Wikipedia biographies, and these differences have been attributed to gender bias [30]. We did not assess the representativeness or achievements of either sex or gender, but this could potentially add a dimension to our analysis in the future.

### Implications of Our Results for the Real World

To answer the question of whether there is a difference between male and female articles on Wikipedia in terms of quality and coverage, we can say there are several differences; however, we must also consider the categorization and the basics of how and when a Wikipedia article is created and why. For example, WikiProject Medicine volunteers who work through a priorities list of missing articles, student initiatives to fill gaps on Wikipedia, individuals who edit Wikipedia with a particular interest in a field, professionals who work in their fields and volunteer on Wikipedia as editors or translators, and others, have determined, based on population’s needs, that these articles were most important. Wikipedia may, therefore, be a mirror of what is required by the general population worldwide rather than a decision of select groups of people or an inherent view. A study conducted in 2021 also found similar results when examining a selected set of professions in terms of gender distribution. It concluded that Wikidata was no more biased than the real world, with men and women being included in similar percentages [56]. Our study has shown that Wikipedia spread of health-related and medical content for female and male categories was similar to the 2 widely used medical textbooks, but with some relative underrepresentation of female centric topics.

More female individuals than males individuals may be seeking health information online. Although the results of this study may be misinterpreted to be more female centric because, it appears that in our sample, there are simply more conditions that have been classified as predominantly female, such as “labiaplasty,” or those exclusively female, such as the “triple X syndrome,” all of which can be due to societal trends or even a rise in medical students who require this information, regardless of their sex. Our sample included conditions such as lung cancer, which accounts for 42% of total deaths in women and 58% of deaths in men in the United States [57,58]. Because of the small difference, this was classified as not sex specific, but on a population level, it is more tilted to male individuals. Therefore, our data must not be viewed in a clear-cut male-female split but contextually.

In the United States, there are 21,570 obstetricians and gynecologists (ob-gyns; 85.2% female individuals; 14.8% male individuals) [59] and 13,780 urologists (69.5% female individuals; 30.5% male individuals) [60], whereby urology treats both sexes [61]. However, it is fair to say that (in the United States in this case) every woman will require at least 1 ob-gyn at some point in her life, but the needs for a urologist are more individual. Ob-gyns specialize in a wide spectrum of situations, which include fertility and hormone disorders, which alone are more numerous than those in urology.

There are several major implications of our research: (1) this study indicates that there may be a difference but not a large disparity between male and female health articles on English-language Wikipedia; (2) this study could be repeated for Wikipedia in other languages, and a comparative analysis could be performed; (3) health information–seeking behavior may be the driving force for the topmost viewed 1000 Wikipedia articles; (4) these data should be interpreted contextually and not be viewed as a simplistic female-male split, especially for non–sex-specific articles in which there may still be disparities in presentation, and understanding the nuances at the population-level data is essential for accurate interpretation; and (5) some health care specializations may be more represented than others on the English-language Wikipedia within the top 1000 most-read health articles, but a wider sample of articles is needed to draw any conclusions.

### Limitations

There is no direct prior work on sex coverage within health Wikipedia articles. We only analyzed a subset of the 1000 most-viewed Wikipedia articles about medicine and health in a snapshot (June 2019) rather than all 50,000 medical articles. This limits the power of our results. The number of male-specific articles within these 1000 articles is low, which is an important finding, but also limits what conclusions can be drawn given the sample size. Non-significant results on some comparisons may reflect insufficient power rather than the absence of any differences. Future research could be conducted using data from the most recent month to compare results and with a larger sample of Wikipedia articles. When viewing the top 1000 articles for January 2023, it was clear that several articles included in our sample had changed their status. For example, the status of many articles has changed from start to class C, and several have risen or fallen in importance or number of views, which can be partly explained by the changes in the COVID-19 pandemic and people’s lifestyles. In addition, our analysis was performed before the COVID-19 pandemic; therefore, it is likely that the coverage of some topics was different from the time after the COVID-19 pandemic.

We have used a methodology previously used in other studies involving Wikipedia articles (eg, [36,48]). One study showed that the quality of Wikipedia articles is affected by the editor’s previous number of edited articles, but not the volume of edits [40]. We could not assess these metrics, which may be interesting to correlate in future studies. Unlike research conducted in 2017 [32], we did not assess the articles using supervised machine learning and, therefore, could not consider metrics that may have introduced other types of biases among contributors and the content itself, such as article and author perspectives, author characteristics, and balance of nonneutral edits. These measurements might have uncovered supplementary information, indicating that both an author’s viewpoints and the article’s prior perspectives played a role in predicting the stance of the resulting edits, with the caveat that these predictors also influence each other [32].

We may not be able to answer the question of whether Wikipedia is biased, but more if the society is biased. For example, with some pages such as monarchs, you would find many more male individuals covered than female individuals, but that is because female individuals were not allowed to have power or these positions. This raises the following question: Is Wikipedia merely reflecting societal trends, and is the Wikipedia community adequately covering what is needed? We could also not assess the degree of risk particular medical conditions pose and the need or urgency of the information for these. Therefore, the strength and limitation of this study is that it captures Wikipedia activity at a specific point in time, but the results are fluid and mirror societal needs. Our analysis identified some gaps and room for improvement in Wikipedia health articles.

Biographies of living persons may not be a fair representation of issues within the core community of Wikipedians (people who edit or contribute to Wikipedia) prioritizing the creation of articles for one sex over the other. Many biographies of living persons end up within Wikipedia because of undisclosed paid editing. An analysis of now-deleted articles created by a collection of accounts from various paid editing companies found that most of the created articles were about male individuals. This leads to the assumption that male individuals and their representatives are more likely to pay money to try to buy an article on Wikipedia than female individuals and their representatives.

### Conclusions

This was the first study to examine the coverage of medical and health content on Wikipedia related to biological sex. In our sample, there were more female health topics than male health topics in the top 1000 most-read health Wikipedia pages in June 2019, but there are relatively fewer female health topics covered than what is seen in medical textbooks. Despite the higher volume of articles on female health topics, the average quality based on various metrics was similar. It is vital to recognize that the needs of people are individual and that both sexes may require or access any of Wikipedia articles. Future endeavors could include prioritizing content to reach GA and FA status as soon as possible. Future research should strive to identify where there may be sex-based differences in needs for health information and whether behavior around health information seeking on Wikipedia differs among the sexes.

## Appendix

Multimedia Appendix 1 A list of 67 sex-specific medical Wikipedia articles and their associated data from views (January 2019) from a list of 1000 most-viewed health Wikipedia articles.

## Abbreviations

CEO: chief executive officer
FA: featured article
Ob-gyn: obstetrics and gynecology
WP: Wikipedia

## Data Availability

All data generated or analyzed during this study are included in this published article and its supplementary information files.
