# Correction: Quality of Male and Female Medical Content on English-Language Wikipedia: Quantitative Content Analysis

**DOI:** 10.2196/66590

**Published:** 2024-09-25

**Authors:** Nuša Farič, Henry WW Potts, James M Heilman

**Affiliations:** 1 School of Informatics University of Edinburgh Edinburgh United Kingdom; 2 Advanced Care Research Center Usher Institute University of Edinburgh Edinburgh United Kingdom; 3 University College London Institute of Health Informatics London United Kingdom; 4 Department of Emergency Medicine Faculty of Medicine University of British Columbia Vancouver, BC Canada

In “Quality of Male and Female Medical Content on English-Language Wikipedia: Quantitative Content Analysis” (J Med Internet Res 2024;26:e47562) an error has been noted.

In the Abstract, the following excerpt:

Of the 1000 Wikipedia health articles, 993 (93.3%) were not sex specific and 67 (6.7%) were sex specific...

has been revised to:

Of the 1000 Wikipedia health articles, 933 (93.3%) were not sex specific and 67 (6.7%) were sex specific...

The correction will appear in the online version of the paper on the JMIR Publications website on September 18, 2024, together with the publication of this correction notice. Because this was made after submission to PubMed, PubMed Central, and other full-text repositories, the corrected article has also been resubmitted to those repositories.

