# Supplementary material for: Quality of Male and Female Medical Content on English-Language Wikipedia: Quantitative Content Analysis
Source: J Med Internet Res. 2024 Sep 12;26:e47562. doi: 10.2196/47562 (PMC11424011; doi:10.2196/47562)
Supplement: Multimedia Appendix 1 [file jmir_v26i1e47562_app1.docx]

Table S1 A list of 67 sex-specific medical WP articles and their associated data from views (January 2019) from a list of 1000 most viewed health WP articles

| **Rank** | **Page title** | **Views** | **Daily average** | **Assessment** | **Importance** | **Classification** | **Bytes** | **Unique refs** | **Total Refs** | **Bytes/**  **Ref** | **Pics** | **Sections** | **Total edits** | **Page watchers** | **Language count** |
| --- | --- | --- | --- | --- | --- | --- | --- | --- | --- | --- | --- | --- | --- | --- | --- |
| 30 | [Endometriosis](https://en.wikipedia.org/wiki/Endometriosis) | [165,818](https://tools.wmflabs.org/redirectviews/?project=en.wikipedia.org&start=2019-06-01&end=2019-06-30&page=Endometriosis) | 5,527 | [B](https://en.wikipedia.org/wiki/Category:B-Class_articles) | [High](https://en.wikipedia.org/wiki/Category:Top-importance_articles) | 1 | 95,430 | 133 | 248 | 385 | 9 | 34 | 2,356 | 301 | 56 |
| 32 | [Stillbirth](https://en.wikipedia.org/wiki/Stillbirth) | [164,123](https://tools.wmflabs.org/redirectviews/?project=en.wikipedia.org&start=2019-06-01&end=2019-06-30&page=Stillbirth) | 5,470 | [C](https://en.wikipedia.org/wiki/Category:C-Class_articles) | [Mid](https://en.wikipedia.org/wiki/Category:Top-importance_articles) | 1 | 44,450 | 44 | 76 | 585 | 6 | 22 | 1,057 | 130 | 39 |
| 79 | [Polycystic ovary syndrome](https://en.wikipedia.org/wiki/Polycystic_ovary_syndrome) | [119,420](https://tools.wmflabs.org/redirectviews/?project=en.wikipedia.org&start=2019-06-01&end=2019-06-30&page=Polycystic_ovary_syndrome) | 3,980 | [B](https://en.wikipedia.org/wiki/Category:B-Class_articles) | [High](https://en.wikipedia.org/wiki/Category:Top-importance_articles) | 1 | 85,232 | 127 | 215 | 396 | 5 | 28 | 2,032 | 333 | 47 |
| 81 | [Human papillomavirus infection](https://en.wikipedia.org/wiki/Human_papillomavirus_infection) | [118,578](https://tools.wmflabs.org/redirectviews/?project=en.wikipedia.org&start=2019-06-01&end=2019-06-30&page=Human_papillomavirus_infection) | 3,952 | [B](https://en.wikipedia.org/wiki/Category:B-Class_articles) | [High](https://en.wikipedia.org/wiki/Category:Top-importance_articles) | 2 | 131,568 | 187 | 306 | 430 | 10 | 40 | 3,958 | 375 | 66 |
| 113 | [Surrogacy](https://en.wikipedia.org/wiki/Surrogacy) | [104,923](https://tools.wmflabs.org/redirectviews/?project=en.wikipedia.org&start=2019-06-01&end=2019-06-30&page=Surrogacy) | 3,497 | [B](https://en.wikipedia.org/wiki/Category:B-Class_articles) | [Low](https://en.wikipedia.org/wiki/Category:Top-importance_articles) | 1 | 68,149 | 79 | 162 | 421 | 8 | 32 | 2,192 | 202 | 53 |
| 125 | [Pregnancy](https://en.wikipedia.org/wiki/Pregnancy) | [101,169](https://tools.wmflabs.org/redirectviews/?project=en.wikipedia.org&start=2019-06-01&end=2019-06-30&page=Pregnancy) | 3,372 | [B](https://en.wikipedia.org/wiki/Category:B-Class_articles) | [Top](https://en.wikipedia.org/wiki/Category:Top-importance_articles) | 1 | 126,554 | 157 | 255 | 496 | 39 | 44 | 6,680 | 572 | 144 |
| 139 | [Female genital mutilation](https://en.wikipedia.org/wiki/Female_genital_mutilation) | [98,121](https://tools.wmflabs.org/redirectviews/?project=en.wikipedia.org&start=2019-06-01&end=2019-06-30&page=Female_genital_mutilation) | 3,270 | [FA](https://en.wikipedia.org/wiki/Category:FA-Class_articles) | [Mid](https://en.wikipedia.org/wiki/Category:Top-importance_articles) | 1 | 161,680 | 333 | 411 | 393 | 29 | 39 | 6,778 | 506 | 86 |
| 179 | [Turner syndrome](https://en.wikipedia.org/wiki/Turner_syndrome) | [88,270](https://tools.wmflabs.org/redirectviews/?project=en.wikipedia.org&start=2019-06-01&end=2019-06-30&page=Turner_syndrome) | 2,942 | [B](https://en.wikipedia.org/wiki/Category:B-Class_articles) | [Mid](https://en.wikipedia.org/wiki/Category:Top-importance_articles) | 1 | 49,809 | 55 | 108 | 461 | 4 | 30 | 2,547 | 255 | 55 |
| 189 | [Abortion](https://en.wikipedia.org/wiki/Abortion) | [86,842](https://tools.wmflabs.org/redirectviews/?project=en.wikipedia.org&start=2019-06-01&end=2019-06-30&page=Abortion) | 2,894 | [B](https://en.wikipedia.org/wiki/Category:B-Class_articles) | [Top](https://en.wikipedia.org/wiki/Category:Top-importance_articles) | 1 | 172,029 | 258 | 336 | 512 | 22 | 30 | 12,817 | 981 | 145 |
| 206 | [Pre-eclampsia](https://en.wikipedia.org/wiki/Pre-eclampsia) | [82,571](https://tools.wmflabs.org/redirectviews/?project=en.wikipedia.org&start=2019-06-01&end=2019-06-30&page=Pre-eclampsia) | 2,752 | [B](https://en.wikipedia.org/wiki/Category:B-Class_articles) | [High](https://en.wikipedia.org/wiki/Category:Top-importance_articles) | 1 | 65,347 | 73 | 203 | 322 | 3 | 25 | 1,338 | 210 | 51 |
| 237 | [Hashimoto's thyroiditis](https://en.wikipedia.org/wiki/Hashimoto%27s_thyroiditis) | [76,986](https://tools.wmflabs.org/redirectviews/?project=en.wikipedia.org&start=2019-06-01&end=2019-06-30&page=Hashimoto%27s_thyroiditis) | 2,566 | [B](https://en.wikipedia.org/wiki/Category:B-Class_articles) | [Mid](https://en.wikipedia.org/wiki/Category:Top-importance_articles) | 2 | 31,544 | 40 | 76 | 415 | 4 | 13 | 808 | 258 | 42 |
| 247 | [Urinary tract infection](https://en.wikipedia.org/wiki/Urinary_tract_infection) | [75,719](https://tools.wmflabs.org/redirectviews/?project=en.wikipedia.org&start=2019-06-01&end=2019-06-30&page=Urinary_tract_infection) | 2,523 | [GA](https://en.wikipedia.org/wiki/Category:GA-Class_articles) | [Top](https://en.wikipedia.org/wiki/Category:Top-importance_articles) | 2 | 64,978 | 94 | 209 | 311 | 8 | 28 | 2,072 | 226 | 55 |
| 262 | [In vitro fertilisation](https://en.wikipedia.org/wiki/In_vitro_fertilisation) | [73,233](https://tools.wmflabs.org/redirectviews/?project=en.wikipedia.org&start=2019-06-01&end=2019-06-30&page=In_vitro_fertilisation) | 2,441 | [B](https://en.wikipedia.org/wiki/Category:B-Class_articles) | [Mid](https://en.wikipedia.org/wiki/Category:Top-importance_articles) | 1 | 137,050 | 177 | 237 | 578 | 5 | 58 | 5,134 | 300 | 53 |
| 299 | [Labiaplasty](https://en.wikipedia.org/wiki/Labiaplasty) | [68,205](https://tools.wmflabs.org/redirectviews/?project=en.wikipedia.org&start=2019-06-01&end=2019-06-30&page=Labiaplasty) | 2,273 | [B](https://en.wikipedia.org/wiki/Category:B-Class_articles) | [Mid](https://en.wikipedia.org/wiki/Category:Top-importance_articles) | 1 | 42,956 | 52 | 86 | 499 | 12 | 17 | 922 | 119 | 20 |
| 320 | [Anorexia nervosa](https://en.wikipedia.org/wiki/Anorexia_nervosa) | [66,313](https://tools.wmflabs.org/redirectviews/?project=en.wikipedia.org&start=2019-06-01&end=2019-06-30&page=Anorexia_nervosa) | 2,210 | [B](https://en.wikipedia.org/wiki/Category:B-Class_articles) | [High](https://en.wikipedia.org/wiki/Category:Top-importance_articles) | 2 | 97,296 | 143 | 239 | 407 | 9 | 33 | 8,334 | 434 | 77 |
| 333 | [Menstrual cycle](https://en.wikipedia.org/wiki/Menstrual_cycle) | [64,895](https://tools.wmflabs.org/redirectviews/?project=en.wikipedia.org&start=2019-06-01&end=2019-06-30&page=Menstrual_cycle) | 2,163 | [FA](https://en.wikipedia.org/wiki/Category:FA-Class_articles) | [High](https://en.wikipedia.org/wiki/Category:Top-importance_articles) | 1 | 81,927 | 126 | 211 | 388 | 16 | 35 | 3,880 | 416 | 67 |
| 353 | [Bulimia nervosa](https://en.wikipedia.org/wiki/Bulimia_nervosa) | [62,622](https://tools.wmflabs.org/redirectviews/?project=en.wikipedia.org&start=2019-06-01&end=2019-06-30&page=Bulimia_nervosa) | 2,087 | [B](https://en.wikipedia.org/wiki/Category:B-Class_articles) | [High](https://en.wikipedia.org/wiki/Category:Top-importance_articles) | 2 | 61,744 | 90 | 164 | 376 | 7 | 20 | 3,898 | 238 | 59 |
| 366 | [Ectopic pregnancy](https://en.wikipedia.org/wiki/Ectopic_pregnancy) | [60,917](https://tools.wmflabs.org/redirectviews/?project=en.wikipedia.org&start=2019-06-01&end=2019-06-30&page=Ectopic_pregnancy) | 2,030 | [B](https://en.wikipedia.org/wiki/Category:B-Class_articles) | [High](https://en.wikipedia.org/wiki/Category:Top-importance_articles) | 1 | 61,067 | 64 | 162 | 377 | 21 | 33 | 1,223 | 190 | 53 |
| 397 | [Caesarean section](https://en.wikipedia.org/wiki/Caesarean_section) | [57,457](https://tools.wmflabs.org/redirectviews/?project=en.wikipedia.org&start=2019-06-01&end=2019-06-30&page=Caesarean_section) | 1,915 | [B](https://en.wikipedia.org/wiki/Category:B-Class_articles) | [High](https://en.wikipedia.org/wiki/Category:Top-importance_articles) | 1 | 106,251 | 142 | 237 | 448 | 21 | 32 | 2,632 | 241 | 72 |
| 399 | [Multiple birth](https://en.wikipedia.org/wiki/Multiple_birth) | [57,381](https://tools.wmflabs.org/redirectviews/?project=en.wikipedia.org&start=2019-06-01&end=2019-06-30&page=Multiple_birth) | 1,912 | [C](https://en.wikipedia.org/wiki/Category:C-Class_articles) | [Mid](https://en.wikipedia.org/wiki/Category:Top-importance_articles) | 1 | 42,979 | 51 | 64 | 672 | 14 | 27 | 1,460 | 151 | 22 |
| 425 | [Triple X syndrome](https://en.wikipedia.org/wiki/Triple_X_syndrome) | [55,873](https://tools.wmflabs.org/redirectviews/?project=en.wikipedia.org&start=2019-06-01&end=2019-06-30&page=Triple_X_syndrome) | 1,862 | [Start](https://en.wikipedia.org/wiki/Category:Start-Class_articles) | [Mid](https://en.wikipedia.org/wiki/Category:Top-importance_articles) | 1 | 13,446 | 11 | 37 | 363 | 6 | 10 | 1,061 | 88 | 35 |
| 461 | [Toxic shock syndrome](https://en.wikipedia.org/wiki/Toxic_shock_syndrome) | [52,402](https://tools.wmflabs.org/redirectviews/?project=en.wikipedia.org&start=2019-06-01&end=2019-06-30&page=Toxic_shock_syndrome) | 1,746 | [B](https://en.wikipedia.org/wiki/Category:B-Class_articles) | [High](https://en.wikipedia.org/wiki/Category:Top-importance_articles) | 2 | 28,124 | 33 | 69 | 408 | 2 | 12 | 812 | 129 | 33 |
| 465 | [Breast cancer](https://en.wikipedia.org/wiki/Breast_cancer) | [52,229](https://tools.wmflabs.org/redirectviews/?project=en.wikipedia.org&start=2019-06-01&end=2019-06-30&page=Breast_cancer) | 1,740 | [B](https://en.wikipedia.org/wiki/Category:B-Class_articles) | [Top](https://en.wikipedia.org/wiki/Category:Top-importance_articles) | 2 | 149,355 | 214 | 295 | 506 | 53 | 42 | 6,550 | 400 | 95 |
| 483 | [Childbirth](https://en.wikipedia.org/wiki/Childbirth) | [51,120](https://tools.wmflabs.org/redirectviews/?project=en.wikipedia.org&start=2019-06-01&end=2019-06-30&page=Childbirth) | 1,704 | [B](https://en.wikipedia.org/wiki/Category:B-Class_articles) | [Top](https://en.wikipedia.org/wiki/Category:Top-importance_articles) | 1 | 134,139 | 151 | 208 | 645 | 29 | 57 | 4,003 | 310 | 101 |
| 559 | [Estrogen](https://en.wikipedia.org/wiki/Estrogen) | [46,953](https://tools.wmflabs.org/redirectviews/?project=en.wikipedia.org&start=2019-06-01&end=2019-06-30&page=Estrogen) | 1,565 | [B](https://en.wikipedia.org/wiki/Category:B-Class_articles) | [High](https://en.wikipedia.org/wiki/Category:Top-importance_articles) | 2 | 55,718 | 85 | 99 | 563 | 7 | 33 | 1,611 | 267 | 75 |
| 573 | [Menstruation](https://en.wikipedia.org/wiki/Menstruation) | [46,044](https://tools.wmflabs.org/redirectviews/?project=en.wikipedia.org&start=2019-06-01&end=2019-06-30&page=Menstruation) | 1,534 | [C](https://en.wikipedia.org/wiki/Category:C-Class_articles) | [Mid](https://en.wikipedia.org/wiki/Category:Top-importance_articles) | 1 | 61,262 | 78 | 125 | 490 | 12 | 23 | 2,087 | 238 | 110 |
| 590 | [Miscarriage](https://en.wikipedia.org/wiki/Miscarriage) | [44,940](https://tools.wmflabs.org/redirectviews/?project=en.wikipedia.org&start=2019-06-01&end=2019-06-30&page=Miscarriage) | 1,498 | [B](https://en.wikipedia.org/wiki/Category:B-Class_articles) | [High](https://en.wikipedia.org/wiki/Category:Top-importance_articles) | 1 | 98,927 | 153 | 342 | 289 | 8 | 45 | 2,090 | 182 | 59 |
| 594 | [Breastfeeding](https://en.wikipedia.org/wiki/Breastfeeding) | [44,836](https://tools.wmflabs.org/redirectviews/?project=en.wikipedia.org&start=2019-06-01&end=2019-06-30&page=Breastfeeding) | 1,494 | [B](https://en.wikipedia.org/wiki/Category:B-Class_articles) | [Top](https://en.wikipedia.org/wiki/Category:Top-importance_articles) | 1 | 157,938 | 233 | 311 | 508 | 41 | 63 | 5,690 | 311 | 85 |
| 611 | [Misoprostol](https://en.wikipedia.org/wiki/Misoprostol) | [44,065](https://tools.wmflabs.org/redirectviews/?project=en.wikipedia.org&start=2019-06-01&end=2019-06-30&page=Misoprostol) | 1,468 | [C](https://en.wikipedia.org/wiki/Category:C-Class_articles) | [Mid](https://en.wikipedia.org/wiki/Category:Top-importance_articles) | 1 | 40,346 | 57 | 73 | 553 | 7 | 15 | 780 | 120 | 28 |
| 657 | [Postpartum depression](https://en.wikipedia.org/wiki/Postpartum_depression) | [42,219](https://tools.wmflabs.org/redirectviews/?project=en.wikipedia.org&start=2019-06-01&end=2019-06-30&page=Postpartum_depression) | 1,407 | [C](https://en.wikipedia.org/wiki/Category:C-Class_articles) | [High](https://en.wikipedia.org/wiki/Category:Top-importance_articles) | 1 | 55,983 | 83 | 175 | 320 | 2 | 26 | 1,426 | 166 | 43 |
| 734 | [Ovarian cancer](https://en.wikipedia.org/wiki/Ovarian_cancer) | [39,590](https://tools.wmflabs.org/redirectviews/?project=en.wikipedia.org&start=2019-06-01&end=2019-06-30&page=Ovarian_cancer) | 1,319 | [B](https://en.wikipedia.org/wiki/Category:B-Class_articles) | [High](https://en.wikipedia.org/wiki/Category:Top-importance_articles) | 1 | 161,101 | 96 | 498 | 323 | 20 | 85 | 2,426 | 131 | 57 |
| 741 | [Menstrual cup](https://en.wikipedia.org/wiki/Menstrual_cup) | [39,339](https://tools.wmflabs.org/redirectviews/?project=en.wikipedia.org&start=2019-06-01&end=2019-06-30&page=Menstrual_cup) | 1,311 | [Start](https://en.wikipedia.org/wiki/Category:Start-Class_articles) | [Low](https://en.wikipedia.org/wiki/Category:Top-importance_articles) | 1 | 36,220 | 50 | 70 | 517 | 7 | 18 | 1,755 | 105 | 44 |
| 753 | [Fetal alcohol spectrum disorder](https://en.wikipedia.org/wiki/Fetal_alcohol_spectrum_disorder) | [38,995](https://tools.wmflabs.org/redirectviews/?project=en.wikipedia.org&start=2019-06-01&end=2019-06-30&page=Fetal_alcohol_spectrum_disorder) | 1,299 | [B](https://en.wikipedia.org/wiki/Category:B-Class_articles) | [High](https://en.wikipedia.org/wiki/Category:Top-importance_articles) | 1 | 93,628 | 90 | 244 | 384 | 4 | 44 | 1,320 | 119 | 36 |
| 758 | [Birth control](https://en.wikipedia.org/wiki/Birth_control) | [38,778](https://tools.wmflabs.org/redirectviews/?project=en.wikipedia.org&start=2019-06-01&end=2019-06-30&page=Birth_control) | 1,292 | [GA](https://en.wikipedia.org/wiki/Category:GA-Class_articles) | [Top](https://en.wikipedia.org/wiki/Category:Top-importance_articles) | 2 | 131,835 | 200 | 323 | 408 | 41 | 32 | 5,095 | 366 | 99 |
| 770 | [Kegel exercise](https://en.wikipedia.org/wiki/Kegel_exercise) | [38,495](https://tools.wmflabs.org/redirectviews/?project=en.wikipedia.org&start=2019-06-01&end=2019-06-30&page=Kegel_exercise) | 1,283 | [C](https://en.wikipedia.org/wiki/Category:C-Class_articles) | [Mid](https://en.wikipedia.org/wiki/Category:Top-importance_articles) | 2 | 14,204 | 24 | 34 | 418 | 5 | 10 | 1,160 | 245 | 30 |
| 779 | [Human chorionic gonadotropin](https://en.wikipedia.org/wiki/Human_chorionic_gonadotropin) | [38,200](https://tools.wmflabs.org/redirectviews/?project=en.wikipedia.org&start=2019-06-01&end=2019-06-30&page=Human_chorionic_gonadotropin) | 1,273 | [C](https://en.wikipedia.org/wiki/Category:C-Class_articles) | [Mid](https://en.wikipedia.org/wiki/Category:Top-importance_articles) | 1 | 45,065 | 56 | 66 | 683 | 8 | 19 | 1,205 | 158 | 41 |
| 784 | [Gravidity and parity](https://en.wikipedia.org/wiki/Gravidity_and_parity) | [38,080](https://tools.wmflabs.org/redirectviews/?project=en.wikipedia.org&start=2019-06-01&end=2019-06-30&page=Gravidity_and_parity) | 1,269 | [C](https://en.wikipedia.org/wiki/Category:C-Class_articles) | [High](https://en.wikipedia.org/wiki/Category:Top-importance_articles) | 1 | 12,158 | 16 | 20 | 608 | 0 | 7 | 245 | 58 | 11 |
| 816 | [Braxton Hicks contractions](https://en.wikipedia.org/wiki/Braxton_Hicks_contractions) | [37,098](https://tools.wmflabs.org/redirectviews/?project=en.wikipedia.org&start=2019-06-01&end=2019-06-30&page=Braxton_Hicks_contractions) | 1,236 | [Start](https://en.wikipedia.org/wiki/Category:Start-Class_articles) | [Low](https://en.wikipedia.org/wiki/Category:Top-importance_articles) | 1 | 4,279 | 4 | 5 | 856 | 1 | 6 | 222 | 45 | 13 |
| 860 | [Rett syndrome](https://en.wikipedia.org/wiki/Rett_syndrome) | [35,985](https://tools.wmflabs.org/redirectviews/?project=en.wikipedia.org&start=2019-06-01&end=2019-06-30&page=Rett_syndrome) | 1,199 | [B](https://en.wikipedia.org/wiki/Category:B-Class_articles) | [Mid](https://en.wikipedia.org/wiki/Category:Top-importance_articles) | 1 | 47,933 | 56 | 128 | 374 | 4 | 20 | 1,075 | 162 | 39 |
| 861 | [Gynaecology](https://en.wikipedia.org/wiki/Gynaecology) | [35,939](https://tools.wmflabs.org/redirectviews/?project=en.wikipedia.org&start=2019-06-01&end=2019-06-30&page=Gynaecology) | 1,197 | [Start](https://en.wikipedia.org/wiki/Category:Start-Class_articles) | [Mid](https://en.wikipedia.org/wiki/Category:Top-importance_articles) | 1 | 19,649 | 18 | 19 | 1034 | 11 | 10 | 1,236 | 154 | 86 |
| 864 | [Progesterone](https://en.wikipedia.org/wiki/Progesterone) | [35,866](https://tools.wmflabs.org/redirectviews/?project=en.wikipedia.org&start=2019-06-01&end=2019-06-30&page=Progesterone) | 1,195 | [B](https://en.wikipedia.org/wiki/Category:B-Class_articles) | [High](https://en.wikipedia.org/wiki/Category:Top-importance_articles) | 2 | 93,965 | 146 | 239 | 393 | 15 | 36 | 1,250 | 178 | 56 |
| 882 | [Preterm birth](https://en.wikipedia.org/wiki/Preterm_birth) | [35,304](https://tools.wmflabs.org/redirectviews/?project=en.wikipedia.org&start=2019-06-01&end=2019-06-30&page=Preterm_birth) | 1,176 | [C](https://en.wikipedia.org/wiki/Category:C-Class_articles) | [High](https://en.wikipedia.org/wiki/Category:Top-importance_articles) | 1 | 117,538 | 165 | 242 | 486 | 10 | 39 | 1,721 | 157 | 37 |
| 892 | [Bacterial vaginosis](https://en.wikipedia.org/wiki/Bacterial_vaginosis) | [35,093](https://tools.wmflabs.org/redirectviews/?project=en.wikipedia.org&start=2019-06-01&end=2019-06-30&page=Bacterial_vaginosis) | 1,169 | [B](https://en.wikipedia.org/wiki/Category:B-Class_articles) | [High](https://en.wikipedia.org/wiki/Category:Top-importance_articles) | 1 | 36,341 | 47 | 105 | 346 | 2 | 14 | 1,223 | 115 | 48 |
| 899 | [Intrauterine device](https://en.wikipedia.org/wiki/Intrauterine_device) | [35,022](https://tools.wmflabs.org/redirectviews/?project=en.wikipedia.org&start=2019-06-01&end=2019-06-30&page=Intrauterine_device) | 1,167 | [B](https://en.wikipedia.org/wiki/Category:B-Class_articles) | [High](https://en.wikipedia.org/wiki/Category:Top-importance_articles) | 1 | 50,136 | 76 | 98 | 512 | 14 | 12 | 690 | 126 | 38 |
| 912 | [Pyelonephritis](https://en.wikipedia.org/wiki/Pyelonephritis) | [34,593](https://tools.wmflabs.org/redirectviews/?project=en.wikipedia.org&start=2019-06-01&end=2019-06-30&page=Pyelonephritis) | 1,153 | [B](https://en.wikipedia.org/wiki/Category:B-Class_articles) | [Mid](https://en.wikipedia.org/wiki/Category:Top-importance_articles) | 2 | 33,904 | 36 | 84 | 404 | 8 | 18 | 512 | 105 | 42 |
| 916 | [Menopause](https://en.wikipedia.org/wiki/Menopause) | [34,523](https://tools.wmflabs.org/redirectviews/?project=en.wikipedia.org&start=2019-06-01&end=2019-06-30&page=Menopause) | 1,150 | [B](https://en.wikipedia.org/wiki/Category:B-Class_articles) | [High](https://en.wikipedia.org/wiki/Category:Top-importance_articles) | 1 | 104,742 | 154 | 250 | 419 | 4 | 39 | 3,168 | 235 | 79 |
| 926 | [Late termination of pregnancy](https://en.wikipedia.org/wiki/Late_termination_of_pregnancy) | [34,243](https://tools.wmflabs.org/redirectviews/?project=en.wikipedia.org&start=2019-06-01&end=2019-06-30&page=Late_termination_of_pregnancy) | 1,141 | [B](https://en.wikipedia.org/wiki/Category:B-Class_articles) | [High](https://en.wikipedia.org/wiki/Category:Top-importance_articles) | 1 | 30,366 | 47 | 62 | 490 | 13 | 12 | 745 | 86 | 4 |
| 961 | [Eating disorder](https://en.wikipedia.org/wiki/Eating_disorder) | [33,213](https://tools.wmflabs.org/redirectviews/?project=en.wikipedia.org&start=2019-06-01&end=2019-06-30&page=Eating_disorder) | 1,107 | [C](https://en.wikipedia.org/wiki/Category:C-Class_articles) | [Mid](https://en.wikipedia.org/wiki/Category:Top-importance_articles) | 2 | 154,912 | 268 | 230 | 674 | 9 | 31 | 4,402 | 209 | 48 |
| 991 | [Mifepristone](https://en.wikipedia.org/wiki/Mifepristone) | [32,363](https://tools.wmflabs.org/redirectviews/?project=en.wikipedia.org&start=2019-06-01&end=2019-06-30&page=Mifepristone) | 1,078 | [B](https://en.wikipedia.org/wiki/Category:B-Class_articles) | [Mid](https://en.wikipedia.org/wiki/Category:Top-importance_articles) | 1 | 98,118 | 136 | 179 | 548 | 7 | 24 | 1,481 | 139 | 27 |
| 992 | [Hysterectomy](https://en.wikipedia.org/wiki/Hysterectomy) | [32,354](https://tools.wmflabs.org/redirectviews/?project=en.wikipedia.org&start=2019-06-01&end=2019-06-30&page=Hysterectomy) | 1,078 | [B](https://en.wikipedia.org/wiki/Category:B-Class_articles) | [High](https://en.wikipedia.org/wiki/Category:Top-importance_articles) | 1 | 70,133 | 100 | 153 | 458 | 11 | 32 | 1,363 | 150 | 36 |
| 137 | [Sildenafil](https://en.wikipedia.org/wiki/Sildenafil) | [98,367](https://tools.wmflabs.org/redirectviews/?project=en.wikipedia.org&start=2019-06-01&end=2019-06-30&page=Sildenafil) | 3,278 | [B](https://en.wikipedia.org/wiki/Category:B-Class_articles) | [Mid](https://en.wikipedia.org/wiki/Category:Top-importance_articles) | 4 | 57,919 | 97 | 127 | 456 | 17 | 33 | 2,844 | 339 | 69 |
| 152 | [Circumcision](https://en.wikipedia.org/wiki/Circumcision) | [93,564](https://tools.wmflabs.org/redirectviews/?project=en.wikipedia.org&start=2019-06-01&end=2019-06-30&page=Circumcision) | 3,118 | [GA](https://en.wikipedia.org/wiki/Category:GA-Class_articles) | [Low](https://en.wikipedia.org/wiki/Category:Top-importance_articles) | 5 | 119,059 | 141 | 324 | 367 | 16 | 38 | 13,516 | 917 | 95 |
| 238 | [Micropenis](https://en.wikipedia.org/wiki/Micropenis) | [76,828](https://tools.wmflabs.org/redirectviews/?project=en.wikipedia.org&start=2019-06-01&end=2019-06-30&page=Micropenis) | 2,560 | [C](https://en.wikipedia.org/wiki/Category:C-Class_articles) | [Mid](https://en.wikipedia.org/wiki/Category:Top-importance_articles) | 5 | 8,774 | 6 | 7 | 1253 | 4 | 7 | 2,083 | 146 | 22 |
| 276 | [Klinefelter syndrome](https://en.wikipedia.org/wiki/Klinefelter_syndrome) | [71,054](https://tools.wmflabs.org/redirectviews/?project=en.wikipedia.org&start=2019-06-01&end=2019-06-30&page=Klinefelter_syndrome) | 2,368 | [B](https://en.wikipedia.org/wiki/Category:B-Class_articles) | [Mid](https://en.wikipedia.org/wiki/Category:Top-importance_articles) | 5 | 42,508 | 64 | 94 | 452 | 7 | 17 | 2,693 | 268 | 52 |
| 315 | [Testosterone](https://en.wikipedia.org/wiki/Testosterone) | [67,126](https://tools.wmflabs.org/redirectviews/?project=en.wikipedia.org&start=2019-06-01&end=2019-06-30&page=Testosterone) | 2,237 | [B](https://en.wikipedia.org/wiki/Category:B-Class_articles) | [High](https://en.wikipedia.org/wiki/Category:Top-importance_articles) | 4 | 127,622 | 197 | 260 | 491 | 16 | 33 | 3,609 | 439 | 80 |
| 417 | [Blue balls](https://en.wikipedia.org/wiki/Blue_balls) | [56,437](https://tools.wmflabs.org/redirectviews/?project=en.wikipedia.org&start=2019-06-01&end=2019-06-30&page=Blue_balls) | 1,881 | [C](https://en.wikipedia.org/wiki/Category:C-Class_articles) | [Low](https://en.wikipedia.org/wiki/Category:Top-importance_articles) | 5 | 5,217 | 8 | 8 | 652 | 2 | 4 | 1,952 | 126 | 13 |
| 442 | [Penis enlargement](https://en.wikipedia.org/wiki/Penis_enlargement) | [54,376](https://tools.wmflabs.org/redirectviews/?project=en.wikipedia.org&start=2019-06-01&end=2019-06-30&page=Penis_enlargement) | 1,812 | [Start](https://en.wikipedia.org/wiki/Category:Start-Class_articles) | [Low](https://en.wikipedia.org/wiki/Category:Top-importance_articles) | 5 | 14,534 | 28 | 36 | 404 | 7 | 10 | 4,461 | 267 | 22 |
| 492 | [Finasteride](https://en.wikipedia.org/wiki/Finasteride) | [50,630](https://tools.wmflabs.org/redirectviews/?project=en.wikipedia.org&start=2019-06-01&end=2019-06-30&page=Finasteride) | 1,687 | [B](https://en.wikipedia.org/wiki/Category:B-Class_articles) | [Mid](https://en.wikipedia.org/wiki/Category:Top-importance_articles) | 4 | 54,474 | 92 | 163 | 334 | 4 | 24 | 1,765 | 174 | 34 |
| 512 | [Tadalafil](https://en.wikipedia.org/wiki/Tadalafil) | [49,681](https://tools.wmflabs.org/redirectviews/?project=en.wikipedia.org&start=2019-06-01&end=2019-06-30&page=Tadalafil) | 1,656 | C | Mid | 4 | 24,950 | 27 | 53 | 471 | 4 | 16 | 1,582 | 188 | 32 |
| 515 | [Color blindness](https://en.wikipedia.org/wiki/Color_blindness) | [49,407](https://tools.wmflabs.org/redirectviews/?project=en.wikipedia.org&start=2019-06-01&end=2019-06-30&page=Color_blindness) | 1,646 | C | Mid | 4 | 84,351 | 91 | 121 | 697 | 13 | 26 | 6,393 | 429 | 79 |
| 528 | [Erectile dysfunction](https://en.wikipedia.org/wiki/Erectile_dysfunction) | [48,535](https://tools.wmflabs.org/redirectviews/?project=en.wikipedia.org&start=2019-06-01&end=2019-06-30&page=Erectile_dysfunction) | 1,617 | [C](https://en.wikipedia.org/wiki/Category:C-Class_articles) | [High](https://en.wikipedia.org/wiki/Category:Top-importance_articles) | 5 | 35,113 | 55 | 88 | 399 | 8 | 17 | 2,132 | 280 | 54 |
| 532 | [Minoxidil](https://en.wikipedia.org/wiki/Minoxidil) | [48,265](https://tools.wmflabs.org/redirectviews/?project=en.wikipedia.org&start=2019-06-01&end=2019-06-30&page=Minoxidil) | 1,608 | [Start](https://en.wikipedia.org/wiki/Category:Start-Class_articles) | [Mid](https://en.wikipedia.org/wiki/Category:Top-importance_articles) | 4 | 22,148 | 30 | 45 | 492 | 5 | 10 | 1,068 | 135 | 30 |
| 765 | [Anabolic steroid](https://en.wikipedia.org/wiki/Anabolic_steroid) | [38,633](https://tools.wmflabs.org/redirectviews/?project=en.wikipedia.org&start=2019-06-01&end=2019-06-30&page=Anabolic_steroid) | 1,287 | [B](https://en.wikipedia.org/wiki/Category:B-Class_articles) | [Mid](https://en.wikipedia.org/wiki/Category:Top-importance_articles) | 4 | 170,519 | 241 | 416 | 410 | 34 | 60 | 6,212 | 268 | 44 |
| 781 | [Inguinal hernia](https://en.wikipedia.org/wiki/Inguinal_hernia) | [38,148](https://tools.wmflabs.org/redirectviews/?project=en.wikipedia.org&start=2019-06-01&end=2019-06-30&page=Inguinal_hernia) | 1,271 | C | Mid | 4 | 30,052 | 29 | 62 | 485 | 14 | 11 | 639 | 117 | 39 |
| 814 | [Vasectomy](https://en.wikipedia.org/wiki/Vasectomy) | [37,213](https://tools.wmflabs.org/redirectviews/?project=en.wikipedia.org&start=2019-06-01&end=2019-06-30&page=Vasectomy) | 1,240 | [B](https://en.wikipedia.org/wiki/Category:B-Class_articles) | [High](https://en.wikipedia.org/wiki/Category:Top-importance_articles) | 5 | 55,505 | 76 | 100 | 555 | 5 | 22 | 2,002 | 148 | 42 |
| 891 | [Prostate cancer](https://en.wikipedia.org/wiki/Prostate_cancer) | [35,102](https://tools.wmflabs.org/redirectviews/?project=en.wikipedia.org&start=2019-06-01&end=2019-06-30&page=Prostate_cancer) | 1,170 | [B](https://en.wikipedia.org/wiki/Category:B-Class_articles) | [Top](https://en.wikipedia.org/wiki/Category:Top-importance_articles) | 5 | 149,092 | 209 | 207 | 720 | 27 | 41 | 4,224 | 319 | 74 |
| 929 | [Priapism](https://en.wikipedia.org/wiki/Priapism) | [34,137](https://tools.wmflabs.org/redirectviews/?project=en.wikipedia.org&start=2019-06-01&end=2019-06-30&page=Priapism) | 1,137 | [Start](https://en.wikipedia.org/wiki/Category:Start-Class_articles) | [Mid](https://en.wikipedia.org/wiki/Category:Top-importance_articles) | 5 | 17,555 | 16 | 69 | 254 | 3 | 18 | 844 | 175 | 42 |

Classification column: 1) "exclusively female", 2) "predominantly female but can also affect males", 3) "not-sex-specific/neutral" (not included in table), 4) "predominantly male but can affect females" and 5) "exclusively male"
